# Pharmacokinetic Evaluation of Tacrolimus in Chinese Adult Patients during the Early Stages Post-Lung Transplantation

**DOI:** 10.3390/jpm13040656

**Published:** 2023-04-11

**Authors:** Yi-Fan Cui, Yan Pan, Min-Fang Zhu, Zheng Jiao

**Affiliations:** 1 Department of Pharmacy, Shanghai Chest Hospital, Shanghai Jiao Tong University School of Medicine, Shanghai 200030, China; 2 School of Basic Medicine and Clinical Pharmacy, China Pharmaceutical University, Nanjing 211198, China; 3 Department of Critical Care Medicine, Shanghai Chest Hospital, Shanghai Jiao Tong University School of Medicine, Shanghai 200030, China

**Keywords:** tacrolimus, lung transplantation, Chinese population, pharmacokinetics, CYP3A4/5 enzyme

## Abstract

Background: Although tacrolimus has been widely used in patients undergoing lung transplantation, few studies have reported the pharmacokinetics of tacrolimus in Chinese patients after lung transplantation. Thus, we aimed to investigate the pharmacokinetics and influential factors in this patient cohort in the early stage after lung transplantation. Methods: We enrolled 14 adult lung transplant recipients who were treated with tacrolimus and then intensively collected blood samples within a 12-h dosing interval. The pharmacokinetic parameters of tacrolimus were calculated using non-compartmental analysis, and the influence of pathophysiological characteristics and CYP3A5*3 and CYP3A4*1G genotypes on the pharmacokinetics of tacrolimus was assessed. Using linear regression analysis, we investigated the correlation between tacrolimus concentration at different sampling points and measured the area under the time-concentration curve (AUC_0–12h_). Results: Geometric mean of apparent clearance (CL/F) was 18.13 ± 1.65 L/h in non-CYP3A5*3/*3 carriers, five times higher than that in CYP3A5*3/*3 carriers (*p* < 0.001). Furthermore, the tacrolimus concentration 4 h after administration had the strongest correlation with AUC_0–12h_ (R^2^ = 0.979). Conclusion: The pharmacokinetics of tacrolimus varied largely between patients during the early stage post-transplantation, which could be partially explained by CYP3A5*3 genetic polymorphisms.

## 1. Introduction

Lung transplantation, followed by postoperative immunosuppressive therapy, is a viable treatment for multiple end-stage pulmonary diseases. Since the 1990s, immunosuppressive therapy has switched from a cyclosporine-based to a tacrolimus-based regimen due to its improved graft survival and reduced rejection rate [1,2,3]. Tacrolimus is a calcineurin inhibitor that prevents interleukin-2 synthesis and subsequently suppresses the T-cell immune response. Currently, it is often combined with mycophenolate mofetil and corticosteroids to prevent further post-transplant rejection [3].

The bioavailability of tacrolimus is generally poor, averaging approximately 25%, and is highly variable, ranging from 5% to 93%, due to pre-systematic metabolism by CYP3A4/5 isoenzymes and P-glycoprotein efflux in the intestine [4,5,6]. Tacrolimus is mainly distributed in the red blood cells (85–95%), and approximately 60% is bound to plasma proteins [7,8]. It is extensively metabolized by CYP3A4/5 enzymes in the liver, and over 95% of its metabolites are excreted in bile. Renal clearance accounts for only 1% of its total clearance [5].

Furthermore, tacrolimus is characterized by a narrow therapeutic index [9,10]; the dose needed to reach a similar exposure can differ by over 10-fold between patients [11]. The large inter-individual variability in tacrolimus pharmacokinetics can be attributed to several factors, such as demographic variables (sex, age, ethnicity), post-transplant time, hepatic impairment, gastrointestinal disturbances, haematocrit and albumin levels, and drug–drug interactions (enzyme inducers or inhibitors) [5,12]. Moreover, CYP3A4/5 genetic polymorphisms are important predictors of the variability in response to tacrolimus [13,14,15,16,17,18]. Studies have shown that CYP3A5*1 carriers require approximately two-fold higher tacrolimus doses to achieve a similar blood concentration as CYP3A5*3/*3 carriers [13,15,19,20].

Current studies have focused on the pharmacokinetic of tacrolimus in patients who underwent kidney and liver transplantation but not lung transplantation. Moreover, compared to other transplant groups, patients who undergo lung transplantation tend to require more frequent adjustments of tacrolimus doses due to the relatively high exposure targets required to prevent rejection and increased co-administered medication that may interfere with the pharmacokinetics of tacrolimus, such as enzyme inhibitor azole-antifungals. In addition, patients who undergo lung transplantation often have co-existing cystic fibrosis and subsequent pancreatic insufficiency, which can lead to poor absorption of tacrolimus [21,22]. Therefore, pharmacokinetic data from other transplant cohorts might not apply to patients undergoing lung transplantation.

Cystic fibrosis is the major underlying disease in Caucasian patients who undergo lung transplantation; however, it has a very low incidence rate in the Chinese population. Furthermore, male and elderly patients account for most of the Chinese lung transplant population and are associated with more complications and risk factors that may affect tacrolimus pharmacokinetics [23,24,25,26,27]. Thus, directly referring to the pharmacokinetic data from studies conducted on Caucasian patients becomes problematic in the Chinese transplant population. Currently, few studies have focused on tacrolimus pharmacokinetics, and little is known about its characteristics in Chinese patients undergoing lung transplants. This prompted the need to investigate the pharmacokinetics of tacrolimus and identify possible influential factors. In addition, previous studies have mainly focused on the stable stages of lung transplantation; however, pharmacokinetic variability may be more pronounced during the early stages post-transplantation when the clinical status and co-administration of medication often change rapidly [28].

Therefore, this study aimed to explore the pharmacokinetic characteristics of tacrolimus and its influencing factors in the early postoperative period in a Chinese population of patients who underwent lung transplantation.

## 2. Material and Methods

### 2.1. Study Design and Population

Adult Chinese patients who underwent lung transplantation between June 2021 and May 2022 at Shanghai Chest Hospital, Shanghai Jiao Tong University School of Medicine were enrolled in this prospective pharmacokinetic study. The inclusion criteria were as follows: patients who (a) underwent lung transplantation within two weeks, (b) were older than 18 years, and (c) were taking tacrolimus as the primary immunosuppressive agent. Patients were excluded from the analysis if they: (a) had intolerable adverse reactions to tacrolimus, (b) received multiple organ transplants, (c) were pregnant or breast-feeding, or (d) had a history of malignant tumours, mental illness, hepatic abnormality, severe gastrointestinal diseases, systemic infection, or any serious condition that might affect tacrolimus pharmacokinetics.

This study was approved by the Ethics Committee of the Shanghai Chest Hospital, and written informed consent was obtained from all patients before enrolment. This study was registered in the Chinese Clinical Trial Registry under the identifier ChiCTR2000036727. All procedures were conducted in accordance with the Helsinki Declaration of 2013. 

After lung transplantation, all patients received a triple immunosuppression regimen of tacrolimus (Prograf^®^; Astellas Ireland Co., Ltd., Killorglin, Ireland), mycophenolate mofetil (MMF; CellCept^®^; Shanghai Roche Pharmaceutical Co., Ltd., Shanghai, China), and corticosteroids that included intravenous methylprednisolone (Solu-Medrol^®^, Pfizer Manufacturing Belgium NV, Puurs, Belgium) or oral prednisone (Tianjin Jinjin Pharmaceutical Co., Ltd., Tianjin, China). In addition, all patients received induction therapy (Basiliximab, Simulect^®^; Novartis Pharma Schweiz AG, Basel, Switzerland) on the first and fourth days after the surgery.

Tacrolimus treatment was initiated at a dose of 0.5 mg every 12 h (6 am and 6 pm) after surgery. The dose was then adjusted by therapeutic drug monitoring to achieve a target whole blood trough concentration between 8 and 12 ng/mL within 3 months after transplantation. Tacrolimus was administered via nasogastric tube immediately after surgery and switched to oral administration after the patients had the endotracheal tube removed.

The initial mycophenolate mofetil oral dose was 1000 mg twice daily and was given along with tacrolimus. The dose was modified based on the clinical response. An intravenous dose of 500 mg (for patients that underwent single lung transplantation) or 750 mg (for patients that underwent bilateral lung transplantation) methylprednisolone was administered during the operation, and then 160 mg was administered every day for the first three days after surgery. The dose was then progressively reduced to 40 mg on the fifth day after surgery, followed by 30 mg of oral prednisone once daily, which was then gradually tapered to a maintenance dose of 5 mg once daily within two weeks. In addition, each patient was orally administered 100 mg voriconazole (Pfizer Italia S.R.L., Latina, Italy) 1 h after taking tacrolimus for invasive aspergillus prophylaxis for three months.

During hospitalisation, patients received a controlled diet served at 7:00 and 17:00 every day to ensure tacrolimus administration in fasting condition or 1–2 h after meals. Patient demographics and clinical data were collected from electronic medical records and included the age, weight, height, body mass index, sex, postoperative time (POT), tacrolimus dosage, concomitant medication, haemoglobin, haematocrit, albumin, total protein, total bilirubin, aspartate aminotransferase, alanine aminotransferase, alkaline phosphatase, γ-glutamyl transpeptidase, blood urea nitrogen, and serum creatinine. Glomerular filtration rate was estimated using the Chronic Kidney Disease Epidemiology Collaboration (CKD-EPI) equation [29].

Serial blood samples (5 mL) were collected in tubes containing ethylenediaminetetraacetic acid (EDTA) before the morning tacrolimus dose (0 h) and at 0.5, 1, 2, 4, 6, 8, 10, and 12 h post-dose at any day within two weeks post-transplantation for each patient. Blood was collected from an indwelling catheter placed in the patient’s forearm vein and stored at −20 °C until analysis.

Tacrolimus whole blood concentrations were determined using a validated liquid chromatography with tandem mass spectrometry (LC-MS/MS) [30]. A ZORBAX SB-C18 column (100 × 2.1 mm, 3.5 μm, Agilent Technologies, Palo Alto, California, USA) was used for chromatographic separation using acetonitrile (solvent A) with 0.1% formic acid aqueous solution and 5 mmol/L ammonium formate (solvent B) as the mobile phase. The flow rate was 0.35 mL/min with an injection volume of 5 μL. The column temperature was set to 40 °C. The lower limit of quantification was 0.5 ng/mL, with a calibration range of 0.5–100 ng/mL. The accuracy and precision of the assay were within 85–115% and <15.0%, respectively.

Genomic DNA was extracted from whole blood samples using an Ezup Column Blood Genomic DNA Purification Kit (Sangon Biotechnology Co., Ltd., Shanghai, China), according to the manufacturer’s protocol. Single nucleotide polymorphisms, including CYP3A5*3 (rs776746) and CYP3A4*1G (rs2242480), were genotyped by independent external contractors (Sangon Biotechnology Co., Ltd., Shanghai, China) using a DNA direct sequencing analyser (Applied Biosystems 3730XL, Foster City, CA, USA). The primer and probe sequences used for genotyping are summarised in Table S1.

Hardy–Weinberg equilibrium was tested using the chi-squared test or Fisher’s exact test, and pairwise D’ and r^2^ values were calculated to estimate linkage disequilibrium (LD) between loci within a gene [31].

### 2.2. Pharmacokinetic Analysis

Non-compartmental analysis was performed using the software package Phoenix WinNonlin (version 8.3, Certara L.P., Princeton, New Jersey, USA) to calculate pharmacokinetic parameters. The dosing interval area under the time-concentration curve (AUC_0–12h_) was estimated using the linear-up log-down trapezoidal method. Apparent clearance (CL/F) was calculated by dividing the tacrolimus dose (mg) by the AUC_0–12h_. The maximal (C_max_) and minimal concentration during the dosing interval (C_min_), as well as the time when C_max_ was achieved (T_max_), were obtained directly from the concentration–time profiles. The AUC_0–12_, C_max_, and C_min_ of dose normalised to 1 mg were then calculated, and all pharmacokinetic parameters were log-transformed before statistical analysis.

The association between blood concentration at each sampling time point and AUC_0–12h_ was also evaluated using linear regression. Abbreviated sampling equations were developed to assess the ability of single concentration–time points to predict the tacrolimus AUC_0–12h_. Predictive performance was evaluated using the coefficients of determination (R^2^), mean prediction error (MPE), and mean absolute prediction error (MAE), which were described by Sheiner and Beal [32] as follows:

(1) $$\mathrm{MPE}=\frac{1}{n}\overset{n}{\underset{i=1}{\sum }}\left({\mathrm{AUC}}_{\mathrm{pred}}-{\mathrm{AUC}}_{\mathrm{obs}}\right)$$

(2) $$\mathrm{MAE}=\frac{1}{n}\overset{n}{\underset{i=1}{\sum }}\left|{\mathrm{AUC}}_{\mathrm{pred}}-{\mathrm{AUC}}_{\mathrm{obs}}\right|$$

where n is the number of patients, and AUC_pred_ and AUC_obs_ refer to the predicted and observed values of AUC_0–12h_ in each patient, respectively.

Next, the |MPE| percentages within 15% (F_15_), 20% (F_20_), and 25% (F_25_) were calculated. The model was considered clinically acceptable if it yielded an MPE ≤ ±15%, MAE ≤ 30%, F_15_ > 40%, F_20_ > 45%, and F_25_ > 50% [33].

All continuous variables were summarised using descriptive statistics. Categorical variables were reported as counts and percentages. The correlation between continuous clinical variables and pharmacokinetic parameters was evaluated using Spearman’s correlation test. Differences in pharmacokinetic parameters or demographic characteristics between individuals with different genotypes were compared using analysis of variance (ANOVA). Comparisons with *p*-values less than 0.05 were considered statistically significant. All statistical analyses were performed using R software (version 3.6.2, https://cran.r-project.org/, accessed on 1 July 2022).

## 3. Results

### 3.1. Study Population

Fourteen patients were enrolled in our study. A total of 125 blood samples were collected at an average of 3.5 days (range 2–8 days) after transplantation and were subsequently analysed. The demographic and clinical characteristics of the lung transplant recipients are summarised in Table 1. Most of the study population was male (85.7%), and 10 patients (71%) were older than 65 years. Chronic obstructive pulmonary disease was the main reason for lung transplantation (78.6%), and no patient had cystic fibrosis.

Five (35.71%) and eight (57.14%) patients exhibited CYP3A5*3/*3 and *1/*3 genotypes, respectively. The CYP3A4*1/*1 and *1/*1G genotypes were observed in nine (64.29%) and four (28.57%) patients, respectively. One CYP3A5*1/*1 homozygous and one CYP3A4*1G/*1G homozygous patient were identified. The CYP3A5 genotype was sub-classified as non-CYP3A5*3/*3 and CYP3A5*3/*3 carriers, and the CYP3A4 genotype was sub-classified as non-CYP3A4*1/*1 and CYP3A4*1/*1 carriers. Genotype distribution followed the Weinberg equilibrium (*p* > 0.05), and a strong LD was observed between CYP3A5*3 (rs776746) and CYP3A4*1G (rs2242480).

All patients received 100 mg voriconazole twice daily for prophylaxis against fungal infection. In addition, two patients were treated for hypertension with amlodipine and nicardipine, and another two patients were treated with amiodarone during the study. No patients experienced graft rejection or severe liver or kidney impairment throughout the study period.

### 3.2. Pharmacokinetic Analysis

**Figure 1:**
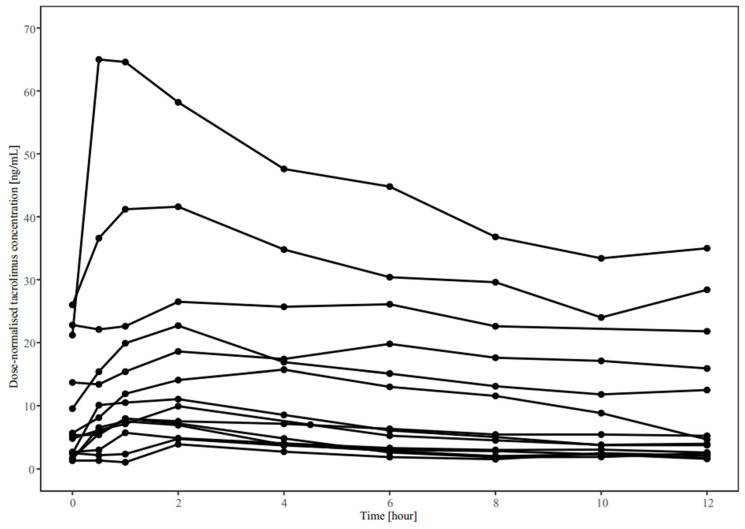
Dose-normalised concentration–time profiles of tacrolimus in 14 adult lung transplant patients.

The time-course profile of tacrolimus concentration over 12 h is presented in Figure 1, while the pharmacokinetic parameters of all 14 patients are shown in Table 2.

**Figure 2:**
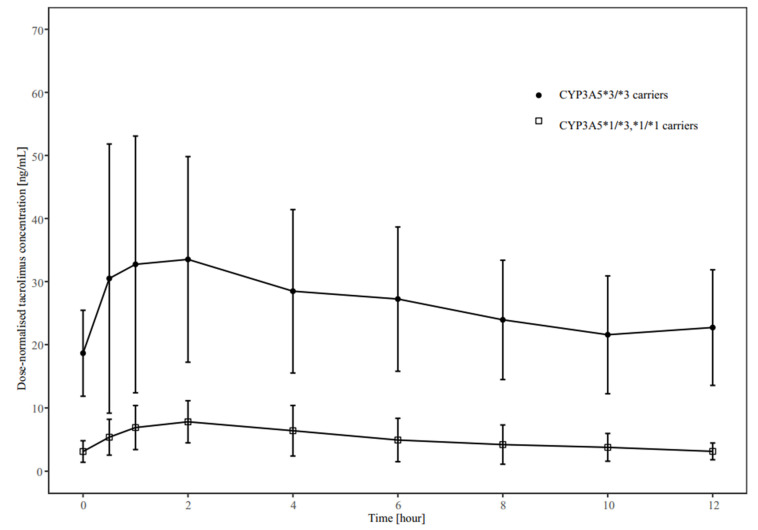
Dose-normalised concentration–time profiles of tacrolimus stratified by CYP3A5 genotype.

The apparent clearance (CL/F), dose-normalised AUC_0–12h_, C_max_, and C_min_ of tacrolimus varied markedly between patients and had a geometric coefficient of variation (CV%) ranging between 69% and 149%. Further, the CYP3A5*3 genotype influenced tacrolimus pharmacokinetics, wherein the dose-normalised tacrolimus concentration in CYP3A5*3/*3 carriers was significantly higher than that in non-CYP3A5*3/*3 carriers (Figure 2). Moreover, non-CYP3A5*3/*3 carriers showed approximately five-fold higher tacrolimus CL/F values than CYP3A5*3/*3 carriers (Table 2). The CV% of CL/F decreased by over 60% in both groups after stratification by CYP3A5 genotype. Additionally, the dose-normalised C_max_ was almost four-fold higher in CYP3A5*3/*3 carriers than in non-CYP3A5*3/*3 carriers (*p* < 0.001). However, there was no significant difference in pharmacokinetic parameters of tacrolimus between CYP3A4 expressers and non-expressers, except for the C_min_ and dose-normalised C_min_ (*p* < 0.05) (Table S2). No significant association was found between other clinical variables and tacrolimus pharmacokinetics.

The relationship between tacrolimus concentration at each time point and the measured AUC_0–12h_ is shown in Table 3. The tacrolimus concentration 4 h after administration (C_4h_) had the strongest correlation with AUC_0–12h_ (R^2^ = 0.979); it also had a good predictive performance for AUC_0–12h_ (MPE = −2%, MAE = 7%, F_15_ = 79%, F_20_ = 93%, and F_25_ = 100%). Furthermore, the concentration at all sampling points in the elimination phase correlated well with the measured AUC_0–12h_ (R^2^ > 0.89). However, the trough concentration (C_0h_) correlated poorly with AUC_0–12h_ (R^2^ = 0.777), but the concentration at 12 h after administration (C_12h_) strongly correlated with AUC_0–12h_ (R^2^ = 0.954).

## 4. Discussion

To the best of our knowledge, this is the first study based on intensive sampling that investigated the pharmacokinetics of tacrolimus in Chinese patients during the early postoperative period following lung transplantation.

Similar to previously published work [18,28], this study demonstrated highly variable tacrolimus pharmacokinetics in the early postoperative period. Our results showed over a 10-fold difference in AUC_0–12h_ between patients taking the same tacrolimus dose. The fluctuation in tacrolimus pharmacokinetics is primarily attributed to the clinical instability of patients during the early postoperative period (within 8 days). The rapid changes in a patient’s physiological and pathological status, along with changes in the administration regimen of concomitant drugs (such as corticosteroids), affect tacrolimus pharmacokinetics and contribute to this instability, which highlights the importance of close monitoring of tacrolimus blood concentrations.

In our study, the median value of T_max_ was 2 h, which was consistent with previous findings in patients soon after lung transplantation [4] and indicated rapid tacrolimus absorption. However, the geometric mean value of CL/F for tacrolimus was 9.95 L/h, which was larger than those in two previous pharmacokinetic studies in Chinese [34,35] and lower than that in Caucasians [36]. A detailed comparison of pharmacokinetics characteristics and patients’ demographics among these studies is presented in Table 4.

Chen et al., reported that the typical CL/F value of tacrolimus was 6.53 L/h, much lower than that in our study after adjusting for weight, tacrolimus daily dose, and haematocrit [34]. However, in their study, the enrolled patients were a median of 86 days post- transplant (ranging from 5 to 937 days). The CL/F of tacrolimus decreased over time after transplantation as the corticosteroid doses were tapered and the haematocrit and albumin levels increased, which may explain this difference [37,38,39]. Additionally, in the study by Chen et al., higher doses of voriconazole were administered (400 mg/d) than those in our study (200 mg/d), which may have enhanced its inhibitory effect on the metabolism of tacrolimus [34,40,41].

In another study conducted by Cai et al., typical tacrolimus CL/F values were reported to be 5.15 L/h for CYP3A5*3/*3 carriers and 6.69 L/h for non-CYP3A5*3/*3 carriers taking voriconazole after adjusting for weight, haematocrit, postoperative time (median 27 days), and tacrolimus daily dose [35]. These values differed from our findings (3.37 L/h for CYP3A5*3/*3 carriers and 18.13 L/h for non-CYP3A5*3/*3 carriers). This discrepancy indicates that in addition to the above-mentioned adjusted factors, there may be some unknown factors affecting the CL/F of tacrolimus. Alternatively, the large inter-individual variability of the interaction between voriconazole and tacrolimus per se could have contributed to this difference. The impact of voriconazole on the tacrolimus pharmacokinetics was influenced by various factors, including the patient’s CYP2C19 genotype, age, degree of liver impairment, and concomitant therapies such as glucocorticoids and proton pump inhibitors [42,43,44]. These factors may have affected voriconazole pharmacokinetics, which ultimately affected the CL/F of tacrolimus.

Darley et al., reported that the CL/F of tacrolimus was 21.6 L/h after adjusting for haematocrit in Caucasian patients taking triazole antifungal agents during the early stages of post-lung transplantation, which was higher than that observed in our study [36]. This discrepancy cannot be explained by differences in CYP3A5 genetic polymorphisms between Chinese and Caucasian populations, as the frequency of poor metabolizers (CYP3A5*3/*3) among Caucasian patients was much higher than among Chinese patients (94% vs. 35.7%, respectively); however, it may be explained by the large number of patients with cystic fibrosis in the Caucasian cohort. CL/F in patients with cystic fibrosis was 87% higher than that in patients without cystic fibrosis because of metabolic disorders caused by liver, pancreatic, and intestinal impairment [1,21]. In addition, the mean age of Chinese patients enrolled in this study was higher than that of Caucasians (67 vs. 44 years), and those patients may have had decreased liver metabolic capacity.

We also explored factors that influence tacrolimus pharmacokinetics in patients during the early stage after lung transplantation. Consistent with previous studies [13,35], we found that the CYP3A5 genotype partially explained the higher inter-individual variability in tacrolimus, with non-CYP3A5*3/*3 carriers having a CL/F more than five times higher than CYP3A5*3/*3 carriers. The difference in CL/F between the two genotypes was much higher than that reported in previous studies (1.2–3 times) [38,45,46,47,48,49], which may be caused by co-therapy of voriconazole in all patients.

Previous studies have shown that the interaction between tacrolimus and triazole antifungal drugs is affected by the CYP3A5 genotype [48,50,51]. CYP3A5 expressers are relatively resistant to the inhibitory effect of voriconazole, whilst the tacrolimus CL/F in CYP3A5 non-expressers decreased further upon co-administration of voriconazole. Therefore, voriconazole co-administration may exacerbate the difference in tacrolimus CL/F between CYP3A5 expressers and non-expressers; however, the impact of CYP3A5 genotype on the interaction between azole antifungal drugs and tacrolimus has not been fully elucidated [35,52,53]. Further investigations are needed to clarify whether the effects of voriconazole on tacrolimus depend on the CYP3A5 genotype.

The relationship between tacrolimus AUC_0–12h_ and concentration at a single sampling time was explored in this study. We observed a strong correlation between tacrolimus concentration 4 h after administration (C_4h_) and AUC_0–12h_ (R^2^ = 0.979), consistent with previous reports [54]. C_4h_ is the point shortly after peak concentration was achieved and may reflect the pharmacokinetic characteristics of the composite absorption, distribution, and metabolism of the drug. Therefore, monitoring the C_4h_ may be a better option due to its ability to predict AUC_0–12h_ more accurately than C_0h_.

Our study has certain limitations that need to be addressed. First, our results were for the early stage post-operation in lung transplant recipients and cannot be extrapolated to the stable stage following lung transplantation. Second, all patients in the study were taking voriconazole, which prevented the proper analysis of the effect of voriconazole on tacrolimus pharmacokinetics in patients with different CYP3A5 genotypes. Finally, the findings of this study should be validated in a larger patient cohort due to its relatively small sample size.

## 5. Conclusions

In summary, this study confirmed the significant impact of the CYP3A5 genotype on tacrolimus pharmacokinetics, which may be enhanced by co-administering voriconazole. Large variations in tacrolimus pharmacokinetics in the early postoperative period after lung transplantation were also observed, highlighting the importance of close therapeutic drug monitoring of tacrolimus in lung transplant patients.

## Figures and Tables

**Table 1:** Patient characteristics.

Variable	Median (Min-Max) /N(%)	Mean ± SD
Number of patients	14 (100%)	
Number of samples	125 (100%)	
Postoperative time (day)	3.50 [2.00, 8.00]	4.14 (1.79)
Tacrolimus dosage(mg)	1 [0.5, 3]	1.32 (0.72)
Sex		
Male (%)	12 (85.7%)	
Female (%)	2 (14.3%)	
Age (years)	67.0 [48.0, 73.0]	65.1 ± 7.67
Weight (kg)	64.5 [46.8, 80.0]	63.0 ± 10.4
Height (cm)	168 [148, 179]	167 ± 8.95
Body Mass Index (kg/m2)	22.5 [16.2, 25.6]	22.5 ± 2.80
Haemoglobin (g/L)	101 [86.0, 122]	102 ±10.8
Haematocrit (%)	30.1 [25.6, 37.0]	30.9 ± 3.22
Albumin (g/L)	44.5 [36.0, 53.0]	44.3 ± 4.86
Total protein (g/L)	62.5 [52.0, 76.0]	62.9 ± 7.28
Total bilirubin (μmol/L)	35.8 [14.8, 116]	39.0 ± 25.2
Aspartate aminotransferase (U/L)	27.5 [19.0, 76.0]	34.2 ± 17.0
Alanine aminotransferase (U/L)	22.5 [8.00, 74.0]	29.2 ± 20.8
Alkaline phosphatase (U/L)	47.5 [39.0, 76.0]	52.4 ± 12.1
γ-glutamyl transpeptidase (U/L)	42.0 [12.0, 135]	48.7 ± 35.4
Blood urea nitrogen (mmol/L)	13.3 [7.50, 26.8]	13.8 ± 5.02
Serum creatinine (μmol/L)	69.5 [44.0, 113]	73.3 ± 23.9
Glomerular filtration rate (mL/min)	97.2 [55.2, 121]	91.4 ± 21.3
Pulmonary diagnosis, n (%)		
Chronic obstructive pulmonary disease	11 (78.6%)	
Pulmonary interstitial fibrosis	3 (21.4%)	
Transplant type, n (%)		
Unilateral	7 (50%)	
Bilateral	7 (50%)	
Genotype (recipients)		
CYP3A5*3 (rs776746)		
*3/*3	5 (35.71%)	
*1/*3	8 (57.14%)	
*1/*1	1 (7.14%)	
CYP3A4*1G (rs2242480)		
*1/*1	9 (64.29%)	
*1/*1G	4 (28.57%)	
*1G/*1G	1 (7.14%)	

**Table 2:** Pharmacokinetic parameters of tacrolimus stratified by CYP3A5 genotype.

Parameter	All (CV%) n = 14	CYP3A5*3/*3 (CV%) n = 5	Non-CYP3A5*3/*3(CV%) n = 9
C_max_(ng/mL)	14.55 ± 1.87 (69.08)	24.05 ± 1.22 (20.35)	11.01 ± 1.83 (66.73) *
C_max_/D(ng/mL)	12.70 ± 2.29 (99.66)	31.74 ± 1.63 (51.92)	7.63 ± 1.54 (45.14) ***
C_min_(ng/mL)	5.58 ± 2.34 (103.17)	12.88 ± 1.38 (32.89)	3.50 ± 1.95 (74.84) **
C_min_/D(ng/mL)	4.87 ± 2.94 (148.55)	17.00 ± 1.48 (40.90)	2.43 ± 1.72 (58.52) ***
T_max_(h)	2.00 (0.50–6.00)	2.00 (0.50–6.00)	2.00 (1.00–4.00)
CL/F(L/h)	9.95 ± 2.60 (121.94)	3.37 ± 1.54 (45.63)	18.13 ± 1.65 (53.32) ***
AUC_0–12h_ (ng·h/mL)	115.23 ± 2.03 (80.50)	224.55 ± 1.22 (20.18)	79.55 ± 1.82 (65.74) **
AUC_0–12h_/D (ng·h/mL)	100.54 ± 2.60 (121.94)	296.30 ± 1.54 (45.63)	55.15 ± 1.65 (53.32) ***

Values are expressed as the geometric mean ± geometric standard deviation or median (minimum-maximum). CV%: geometric coefficient of variation; C_max_: maximum concentration; C_max_/D: C_max_ of dose normalised to 1 mg; C_min_: minimum concentration; C_min_/D: C_min_ of dose normalised to 1 mg; T_max_: time at which C_max_ occurred; CL/F: apparent clearance; AUC_0–12h_: AUC within 12-h dosing interval; AUC_0–12h_/D: AUC_0–12h_ of dose normalised to 1 mg. * *p* ≤ 0.05, ** *p* ≤ 0.01 and *** *p* ≤ 0.001 (ANOVA) Non-CYP3A5*3/*3 carriers versus CYP3A5*3/*3 carriers.

**Table 3:** The predictive performance of the single-point sampling strategy for AUC_0–12h_.

Sampling Time (h)	Geometric Mean ± Geometric SD	R^2^	MPE (%) Median (Range)	MAE (%) Median (Range)	>15% ^a^	>20% ^b^	>25% ^c^
0	6.08 ± 2.24	0.777	11 (−36–119)	18 (1–119)	8	7	5
0.5	9.68 ± 2.45	0.791	0 (−31–78)	22 (3–78)	8	7	6
1	11.56 ± 2.47	0.748	−1 (−32–72)	12 (0–72)	7	5	5
2	13.86 ± 1.89	0.869	−3 (−22–43)	19 (2–43)	8	7	2
4	11.16 ± 2.01	0.979	−2 (−24–16)	7 (1–24)	3	1	0
6	9.10 ± 2.15	0.970	−1 (−24–35)	7 (2–35)	4	4	2
8	7.74 ± 2.20	0.945	2 (−34–44)	11 (1–44)	5	5	4
10	6.98 ± 1.94	0.892	3 (−28–97)	14 (3–97)	5	4	3
12	6.76 ± 2.30	0.954	0 (−24–105)	8 (1–105)	4	3	2

R^2^: coefficient of determination; MPE (%): mean prediction error; MAE (%): mean absolute prediction error. a: number of cases exceeding ±15%; b: number of cases exceeding ±20%; c: number of cases exceeding ±25%.

**Table 4:** Comparison of pharmacokinetics characteristics and patients’ demographics among different studies.

First Author (Year of Publication)	Country	Number of Patients (NCF/CF)	Number of Patients Taking Azole Antifungal	Postoperative Time (Day)	Daily Dose of Tacrolimus (mg)	Sampling Time	Age (Year)	Weight (kg)	Haematocrit (%)	Adjusted CL/F * (L/h)
Darley, et al., (2019) [36]	Australia	18 (13/5)	15	21	8.0 (4.0–11.2)	pre-dose, 1, 2, 3, 4, 5, and 6 h post-dose	44 ± 16	62 ± 15	/	21.6
Cai, et al., (2020) [35]	China	52 (52/0)	37	27 (2–162)	3 (0.125–13)	pre-dose	54 (16–78)	55 (32–75)	29.3 (18–41.7)	5.15
Chen, et al., (2022) [34]	China	52 (52/0)	52	86 (5–937)	0.75 (0.17–4)	pre-dose	60 (17–70)	50 (32.0–86.4)	31.5 (17.0–48.0)	6.53
Current study	China	14 (14/0)	14	3.5 (2–8)	2 (1.0–6)	pre-dose, 0.5, 1, 2, 4, 6, 8, 10, and 12 h post-dose	67 (48–73)	64.5 (46.8–80.0)	30.1 (25.6–37.0)	9.95

The data are presented as median (range) or mean ± SD. *: Adjust the CL to a typical patient with 64.5-kg, daily dose of 2 mg, haematocrit of 30%, and 3.5 days post-transplantation. CL/F: apparent clearance; CF: patients with cystic fibrosis; NCF: patients without cystic fibrosis. Darley, et al. [36]: CL/F = 14.4/(HCT/45). Chen, et al. [34]: CL/F = 3.7 × (WT/70)^0.75^ × (VOZ/2.02)^−0.288^ × (DD/0.75)^0.618^ × (HCT/31.5)^−0.511^. Cai, et al. [35]: CL/F = 13.1 × (WT/70)^0.75^ × (HCT/30)^−0.868^ × (DD/3)^0.616^ × (POT/30)^0.0807^ × 1.3 (if CYP3A5*1 carriers) × 0.638 (if with voriconazole comedication). WT: weight, VOZ: voriconazole trough concentrations, DD: tacrolimus daily dose, HCT: haematocrit, POT: postoperative time.

## Data Availability

The datasets generated during and/or analysed during the current study are available from the corresponding author on reasonable request.

## Supplementary Materials

The following supporting information can be downloaded at: https://www.mdpi.com/article/10.3390/jpm13040656/s1, Table S1: PCR primer sequences used to distinguish between the CYP3A5*3 and CYP3A4*1G genotypes; Table S2: Pharmacokinetic parameters of tacrolimus stratified by CYP3A4 genotype.
