# Supplementary material for: Pharmacokinetic Evaluation of Tacrolimus in Chinese Adult Patients during the Early Stages Post-Lung Transplantation
Source: J Pers Med. 2023 Apr 11;13(4):656. doi: 10.3390/jpm13040656 (PMC10145266; doi:10.3390/jpm13040656)
Supplement: Supplementary file 1 [file jpm-13-00656-s001.zip › jpm-2232629-supplementary.pdf]

Table S1 PCR primer sequences used to distinguish between the CYP3A5*3 and CYP3A4*1G genotypes.

Gene	Primer
CYP3A5*3 (rs776746)	Forward：5′ TAATCCATACCCCTAGTTGTACGAC 3′
	Reverse：5′ TACCTGCCTTCAATTTTTCACTG 3′
CYP3A4*1G (rs2242480)	Forward：5′ CTTTTCAGAGCCTTCCTACATAGAG 3′
	Reverse：5′ GATGCCCTACATTGATCTGATTTAC 3′

Table S2 Pharmacokinetic parameters of tacrolimus stratified by CYP3A4 genotype .

Parameter	All (CV%) n=14	CYP3A4*1/*1 (CV%) n=9	Non-CYP3A4*1/*1(CV%) n=5
C_{max}(ng/mL)	14.55±1.87 (69.08)	17.84±1.68 (55.77)	10.08±1.98 (76.94)
C_{max}/D(ng/mL)	12.70±2.29 (99.66)	17.06±2.29 (99.58)	7.46±1.75 (60.85)
C_{min}(ng/mL)	5.58±2.34 (103.17)	7.82±2.15 (88.96)	3.03±1.98 (77.34)*
C_{min}/D(ng/mL)	4.87±2.94 (148.55)	7.48±2.88 (143.94)	2.25±1.85 (67.96)*
T_{max}(h)	2.00 (0.50-6.00)	2.00 (0.50-6.00)	2.00 (1.00-4.00)
CL/F(L/h)	9.95±2.60 (121.94)	6.97±2.55 (118.70)	18.85±1.89 (70.97)
AUC_{0-12h} (ng·h/mL)	115.23±2.03 (80.50)	150.02±1.89 (70.72)	71.67±1.84 (67.36)
AUC_{0-12h}/D(ng·h/mL)	100.54±2.60 (121.94)	143.41±2.55 (118.70)	53.05±1.89 (70.97)

Values are expressed as the geometric mean ± geometric standard deviation or median (minimum-maximum). CV%: geometric coefficient of variation; C_{max}: maximum concentration;C_{max}/D: C_{max} of dose normalised to 1mg; C_{min}: minimum concentration; C_{min}/D: C_{min} of dose normalised to 1mg; T_{max}: time at which C_{max} occurred; CL/F: apparent clearance; AUC_{0-12h} : AUC within 12-hour dosing interval; AUC_{0-12h}/D: AUC_{0-12h} of dose normalised to 1mg.

*$p \leq 0.05$ (ANOVA) Non- CYP3A4*1/*1 carriers versus CYP3A4*1/*1 carriers
